# Supplementary material for: Induction, decay, and determinants of functional antibodies following vaccination with the RTS,S malaria vaccine in young children
Source: BMC Med. 2022 Aug 25;20:289. doi: 10.1186/s12916-022-02466-2 (PMC9402280; doi:10.1186/s12916-022-02466-2)
Supplement: Supplementary file 1 — Additional file 1: Figure S1. Comparator vaccine does not induce antibodies that interact with FcγRs. Figure S2. FcγR-binding efficiencies of antibodies to the NANP-repeat and C-terminal regions of CSP. Figure S3. Induction of FcγR-binding antibodies to the central-repeat and C-terminal regions of CSP in younger and older children. Figure S4. RTS,S vaccine-induced FcγR-binding antibodies are negatively correlated with malaria exposure. Figure S5. Kinetics of RTS,S vaccine-induced functional antibodies over time. Figure S6. Kinetics of RTS,S vaccine-induced IgG magnitude over time. Figure S7. Kinetics of RTS,S vaccine-induced IgG to the NANP and CT regions over time. Figure S8. Decay of IgG1 and IgG3 relative to total IgG. Table S1. Spearman’s correlation coefficients between immune parameters that relate to FcγRIIa and FcγRIII binding among samples tested at M3. Table S2. Estimated half-life for IgG and functional factors. Table S3. Associations between log IgG subclass, log functional antibodies and time. Table S4-5. Associations between FcγRIIa and FcγRIII with IgG subclass. Table S6-7. Associations between FcγRIIa, FcγRIII, and IgG to NANP and IgG to CT. Table S8-10. Associations between opsonic phagocytosis by neutrophils with IgG subclass, IgG to NANP and IgG to CT, FcγRIII and FcγRIIa binding. Table S11. Associations between opsonic phagocytosis by THP-1 cells and IgG subclass. [file 12916_2022_2466_MOESM1_ESM.docx]

ADDITIONAL FILE 1

**Induction, decay, and determinants of functional antibodies following vaccination with the RTS,S malaria vaccine in young children**

Gaoqian Feng^1 ,2^, *Liriye Kurtovic^1,3^, *Paul A. Agius^1,4,5^, Elizabeth Aitken^6^, Jahit Sacarlal ^7,8^, Bruce Wines^1,3,9^, P. Mark Hogarth^1,3,9^, Stephen Rogerson^2,6^, Freya J. I. Fowkes^1,4,5^, Carlota Dobaño^7,10^, James G. Beeson^1,2,11,^

*1. Burnet Institute, Melbourne, Australia.*

*2. Department of Medicine, The University of Melbourne, Melbourne, Australia*

*3. Central Clinical School, Monash University, Melbourne, Australia*

*4. Department of Epidemiology and Preventative Medicine, Monash University, Melbourne, Australia*

*5. Melbourne School of Population and Global Health, The University of Melbourne, Melbourne, Australia*

*6. Peter Doherty Institute, The University of Melbourne, Melbourne Australia*

*7. Centro de Investigação em Saúde de Manhiça, Maputo, Mozambique.*

*8. Faculdade de Medicina, Universidade Eduardo Mondlane (UEM), Maputo, Mozambique.*

*9. Department of Pathology, The University of Melbourne, Melbourne, Australia*

*10. ISGlobal, Hospital Clínic Universitat de Barcelona, Barcelona, Catalonia, Spain.*

*11. Department of Microbiology, Monash University, Clayton, Australia.*

*These authors contributed equally to this work

**Figure S1.** **Comparator vaccine does not induce antibodies that interact with FcγRs.**

Serum samples from children in the comparator vaccine group from Manhiça (blue box plots, n=25) and Ilha Josina (orange box plots, n=24) study sites were tested for FcγRIIa (left panels) and FcγRIII-binding (right panels). Samples collected at baseline (month 0, M0) and after the third vaccination (month 3, M3) were tested against (**A-B**) full-length CSP, and the (**C-D**) NANP-repeat (NANP) and (**E-F**) C-terminal (CT) regions of CSP. Data from the RTS,S vaccine group at M3 are shown for comparison. Boxes represents the 75th percentile, median, and 25th percentile and whiskers represent the highest and lowest values within 1.5× IQR. Data are shown as the OD at 450nm on the Y-axis and the percentage of children with a positive response are indicated, and reactivity within the comparator groups (M0 vs. M3) were compared by Wilcoxons tests and reactivities between the RTS,S group and the comparator group at M3 were compared by Kruskal–Wallis test.

**Figure S2. FcγR-binding efficiencies of antibodies to the NANP-repeat and C-terminal regions of CSP.**

Samples collected after RTS,S vaccination (at M3) were evaluated for FcγRIIa **(A)** and FcγRIII **(B)** binding efficiencies (n=75). FcγR-binding efficiency was calculated as the ratio of FcγR-binding relative to IgG reactivity, specific to the NANP-repeat (NANP) or C-terminal (CT) regions of CSP. FcγR-binding efficiencies between the NANP and CT regions were compared using the Wilcoxon matched-paired signed rank test, p<0.001) Individuals with low IgG binding to the CT region or NANP repeat region (bottom quartile) were excluded from analysis.

**Figure S3. Induction of FcγR-binding antibodies to the central-repeat and C-terminal regions of CSP in younger and older children.**

Serum samples from children in the RTS,S vaccine group were stratified into younger (12-24 months) and older (24-60 months) age groups from Manhiça (blue box plots; n=11 and n=39, respectively) and Ilha Josina (orange box plots; n=24 and n=26, respectively) study sites. Samples collected after vaccination were tested for (**A**) FcγRIIa and (**B**) FcγRIII-binding to the central-repeat (NANP) and C-terminal (CT) regions of CSP. Boxes represent the interquartile range (IQR) with median (bar) and whiskers represent the highest and lowest values within 1.5× IQR. The percentage of children with a positive response are shown. P values were calculated using the Kruskal-Wallis test.

**Figure S4. RTS,S vaccine-induced FcγR-binding antibodies are negatively correlated with malaria exposure.**

Serum samples from children vaccinated with RTS,S from the Ilha Josina study site (n=49) were tested for FcγRIIa and FcγRIII-binding to CSP and for IgG to merozoite antigens MSP2 which are established biomarkers of malaria exposure. The correlation between IgG to MSP2 and **(A)** FcγRIIa or (**B**) FcγRIII were plotted. Individual samples are shown as scatter plots and correlations were evaluated using Spearman’s correlation coefficient (r). X-axis and Y-axis values are optical density.

**Figure S5. Kinetics of RTS,S vaccine-induced functional antibodies over time.**

A subset of children in the RTS,S vaccine group from the Manhiça study site were followed up over 5 years post vaccination. Serum samples collected at baseline (M0), 30 days after vaccination (M3) and later time points (M8.5, M21, M33, M45 and M63) were tested for (**A**) opsonic phagocytosis by neutrophils (n=30), (**B**) opsonic phagocytosis by THP-1 cells (n=30), (**C**) antibody dependent respiratory burst (ADRB, n=30)), (**D**) FcγRIIa binding to CSP (n=52 for M0-M3, n=30 for M8.5-M63), (**E**) FcγRIIa binding to NANP repeats (n=52 for M0-M3, n=30 for M8.5-M63), (**F**) FcγRIIa binding to CT (n=52 for M0-M3, n=30 for M8.5-M63), (**G**) FcγRIII binding to CSP (n=52 for M0-M3, n=30 for M8.5-M63), (**H**) FcγRIII binding to NANP repeats (n=52 for M0-M3, n=30 for M8.5-M63), (**I**) FcγRIII binding to CT (n=52 for M0-M3, n=30 for M8.5-M63). Boxes represent the interquartile range (IQR) with median (bar) and whiskers represent the highest and lowest values within 1.5× IQR.

**Figure S6. Kinetics of RTS,S vaccine-induced IgG magnitude over time.**

A subset of children in the RTS,S vaccine group from the Manhiça study site were followed up over 5 years post vaccination. Serum samples collected at baseline (M0, n=52), 30 days after vaccination (M3, n=52) and later time points (M8.5, M21, M33, M45 and M63, n=30) were tested for **(A)** IgG binding to CSP, **(B)** IgG binding to NANP repeats **(C)** IgG binding to CT region. IgG subclasses to CSP were also tested, including **(D)** IgG1, **(E)** IgG2, **(F)** IgG3 and **(G)** IgG4 were also tested. Boxes represent the interquartile range (IQR) with median (bar) and whiskers represent the highest and lowest values within 1.5× IQR.


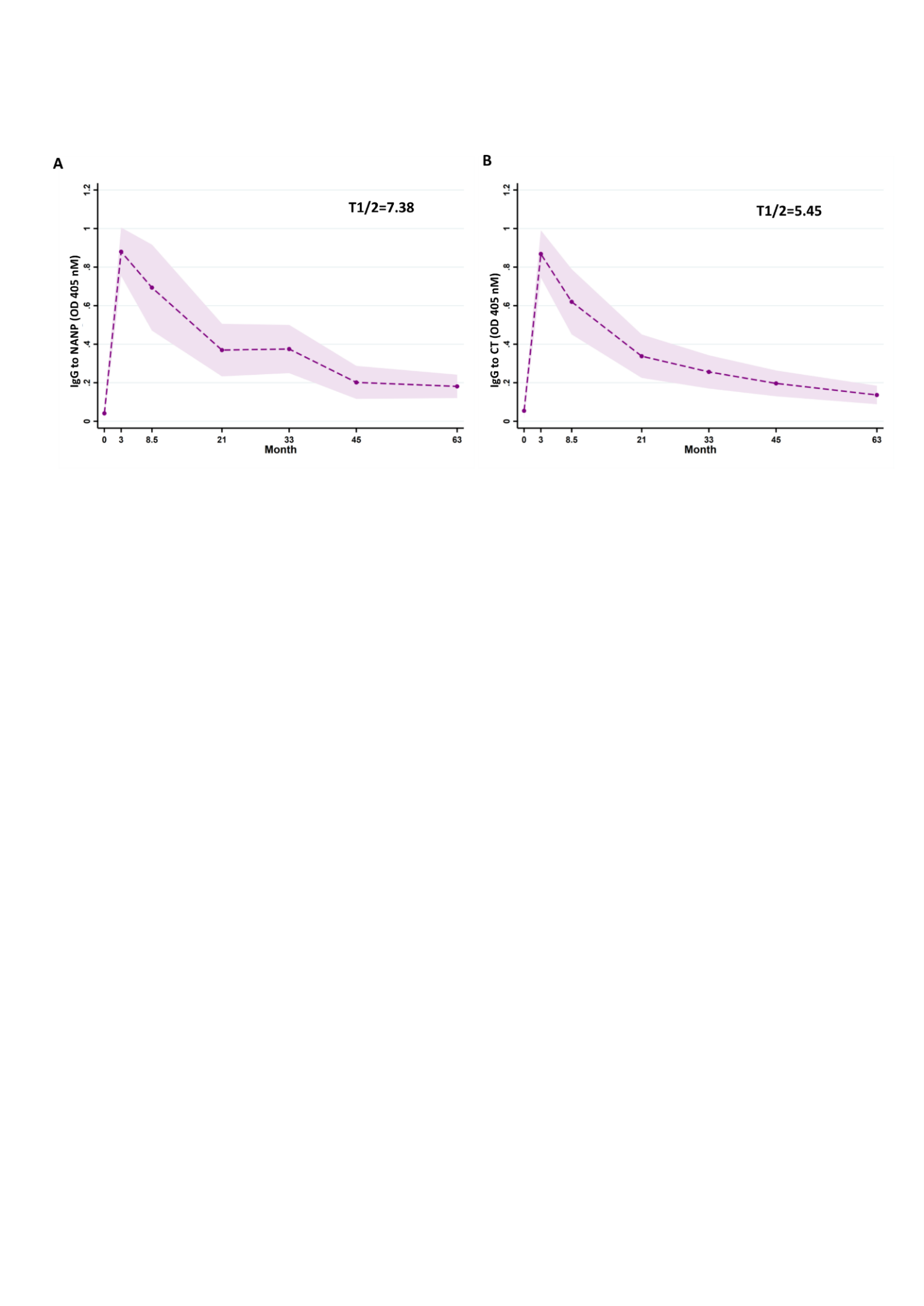


**Figure S7. Kinetics of RTS,S vaccine-induced IgG to the NANP and CT regions over time.**

A subset of children in the RTS,S vaccine group from the Manhiça study site were followed up over 5 years post vaccination. Serum samples collected at baseline (M0, n=52), 30 days after vaccination (M3, n=52) and later time points (M8.5, M21, M33, M45 and M63, n=30) were tested for **(A)** IgG binding to NANP repeats **(B)** IgG binding to CT region. The dashed lines represent the predicted means, the shaded area represent the 95% CIs and “T1/2” indicates the estimated half-lives from the generalized linear mixed model (GLMM). Distributions of actual individual data are presented in Additional file 1: Figure S5, S6 and details of the half-lives are presented in Additional file 1: Table S2.

**Figure S8. Decay of IgG1 and IgG3 relative to total IgG.**

Among children in the RTS,S vaccine group from the Manhiça study site (n=30), serum samples collected 30 days after vaccination (M3) and later time points (M8.5 and M21) were tested for total IgG, IgG1 and IgG3 to CSP. The ratio of (**A**) IgG1 to total IgG (n=26, Friedman test, p<0.001), (**B**) IgG3 to total IgG (n=26, Friedman test, p<0.001), and (**C**) IgG3 to IgG1 (n=21, Friedman test, p=0.002) were shown. Dots represent each individual value, black lines and blue shade represent the mean of 95% CI from each time point.

**Table S1. Spearman’s correlation coefficients between immune parameters that relate to FcγRIIa and FcγRIII binding among samples tested at M3**

|  | FcγRIIa to CSP | FcγRIII to CSP | FcγRIIa to NANP | FcγRIII to NANP | FcγRIIa to CT | FcγRIII to CT | IgG to CSP | IgG to NANP | IgG to CT | IgG1 to CSP | IgG2 to CSP |  |
| --- | --- | --- | --- | --- | --- | --- | --- | --- | --- | --- | --- | --- |
| FcγRIII to CSP | 0.934 |  |  |  |  |  |  |  |  |  |  |  |
| FcγRIIa to NANP | 0.547 | 0.619 |  |  |  |  |  |  |  |  |  | 1 |
| FcγRIII to NANP | 0.663 | 0.704 | 0.938 |  |  |  |  |  |  |  |  | 0.9 |
| FcγRIIa to CT | 0.895 | 0.908 | 0.521 | 0.622 |  |  |  |  |  |  |  | 0.8 |
| FcγRIII to CT | 0.920 | 0.958 | 0.550 | 0.656 | 0.944 |  |  |  |  |  |  | 0.7 |
| IgG to CSP | 0.891 | 0.871 | 0.682 | 0.762 | 0.871 | 0.877 |  |  |  |  |  | 0.6 |
| IgG to NANP | 0.794 | 0.775 | 0.472 | 0.578 | 0.850 | 0.831 | 0.785 |  |  |  |  | 0.5 |
| IgG to CT | 0.590 | 0.658 | 0.865 | 0.898 | 0.581 | 0.620 | 0.722 | 0.556 |  |  |  | 0.4 |
| IgG1 to CSP | 0.839 | 0.848 | 0.611 | 0.713 | 0.790 | 0.838 | 0.877 | 0.690 | 0.661 |  |  | 0.3 |
| IgG2 to CSP | 0.517 | 0.435 | 0.337 | 0.356 | 0.654 | 0.498 | 0.576 | 0.645 | 0.309 | 0.432 |  |  |
| IgG3 to CSP | 0.689 | 0.720 | 0.413 | 0.458 | 0.686 | 0.710 | 0.610 | 0.588 | 0.393 | 0.555 | 0.419 |  |

Notes: n=98; a. All correlation coefficients are significant (p<0.001). Correlations for IgG4 are not included because the magnitude of IgG4 was low and all correlations were non-significant and IgG4 has limited activity for FcγR binding. IgM was not included because it does not bind FcγRs

**Table S2: Estimated half-life (months) for IgG and functional factors: Half-life (*t_1/2_*), standard error (s.e.), 95% confidence interval (95% CI) and probability value (p-value) from** **generalised linear mixed modelling.**

| *Outcome* | | *t_1/2_^a^* (s.e.) | | 95% CI | | p-value | |
| --- | --- | --- | --- | --- | --- | --- | --- |
|  | |  | |  | |  | |
| IgG1(n=52) | | 4.4 (.75) | | 2.93,5.87 | | <.001 | |
|  | |  | |  | |  | |
| IgG2 (n=52) | | 4.59 (1.80) | | 1.07,8.11 | | .011 | |
|  | |  | |  | |  | |
| IgG3 (n=52) | | 2.91 (.40) | | 2.13,3.69 | | <.001 | |
|  | |  | |  | |  | |
| IgG4 (n=52) | | 7.51 (6.1) | | -4.54,19.6 | | .222 | |
|  | |  | |  | |  | |
| IgG to CSP (n=52) | | 17.3 (1.81) | | 13.7,20.8 | | <.001 | |
|  | |  | |  | |  | |
| IgG to NANP (n=52) | | 7.38 (.38) | | 6.62,8.15 | | <.001 | |
|  | |  | |  | |  | |
| IgG to CT (n=52) | | 5.45 (.99) | | 3.52,7.39 | | <.001 | |
|  | |  | |  | |  | |
| Neutrophil ADRB (n=30) | | 2.14 (.65) | | .88,3.42 | | .001 | |
|  | |  | |  | |  | |
| Neutrophil Phagocytosis (n=33) | | 18.1 (.13) | | 17.8,18.36 | | <.001 | |
|  | |  | |  | |  | |
| Neutrophil THP1 (n=30) | | 17 (1.80) | | 13.5,20.5 | | <.001 | |
|  | |  | |  | |  | |
| FcγRIII (n=52) | | 2.21 (.35) | | 1.53,2.89 | | <.001 | |
|  | |  | |  | |  | |
| FcγRIIa (n=50) | | 2.82 (.47) | | 1.90,3.74 | | <.001 | |
|  | |  | |  | |  | |

^a^ Estimated months for the parameter to decay to half its peak value. Linear splines were used to model the functional form of time. These GLMM’s applied a random intercept for study participant, random slope for 0-3 months with an independent covariance structure for the random effects. Post-estimation non-linear equations were used to estimate half-life from respective latent growth curve models. All parameters were quantified using responses to full length CSP, except IgG to NANP repeat and CT domain as indicated.

**Table S3: Associations between log IgG subclass, log functional antibodies and time (per month): Rate ratios regression (RR), standard error (s.e.), 95% confidence interval (95% CI) and probability value (p-value) from generalised linear mixed modelling.**

| *Outcome* | | RR ^a^ (s.e.) | | 95% CI | | p-value ^b^ | |
| --- | --- | --- | --- | --- | --- | --- | --- |
|  | |  | |  | |  | |
| IgG1 (n=52) | |  | |  | |  | |
| *≤ 3-months* | | 2.56 (0.42) | | 1.85,3.54 | | <0.001 | |
| *3-8.5 months* | | 0.85 (0.02) | | 0.81,0.90 | | <0.001 | |
| *8.5-33 months* | | 0.97 (0.01) | | 0.96,0.98 | | <0.001 | |
| *33+ months* | | 0.99 (0.01) | | 0.98,1.00 | | 0.111 | |
|  | |  | |  | |  | |
| IgG2 (n=52) | |  | |  | |  | |
| *≤ 3-months* | | 1.77 (0.24) | | 1.37,2.30 | | <0.001 | |
| *3-8.5 months* | | 0.86 (0.05) | | 0.77,0.97 | | 0.011 | |
| *8.5-33 months* | | 0.91 (0.01) | | 0.89,0.94 | | <0.001 | |
| *33-45 months* | | 1.08 (0.05) | | 0.98,1.19 | | 0.110 | |
| *45+ months* | | 1.00 (0.03) | | 0.96,1.06 | | 0.732 | |
|  | |  | |  | |  | |
| IgG3 (n=52) | |  | |  | |  | |
| *≤ 3-months* | | 2.09 (0.25) | | 1.64,2.65 | | <0.001 | |
| *3-8.5 months* | | 0.79 (0.03) | | 0.74,0.84 | | <0.001 | |
| *8.5-45 months* | | 0.97 (0.01) | | 0.95,0.98 | | <0.001 | |
| *45+ months* | | 1.04 (0.03) | | 0.98,1.10 | | 0.226 | |
|  | |  | |  | |  | |
| IgG4 (n=52) | |  | |  | |  | |
| *≤ 3-months* | | 1.52 (0.45) | | 0.85,2.71 | | 0.160 | |
| *3-8.5 months* | | 1.12 (.16) | | 0.85,1.47 | | 0.424 | |
| *8.5-21 months* | | 1.06 (0.07) | | 9.93,1.21 | | 0.372 | |
| *21-33 months* | | 0.91 (0.07) | | 9.79,1.06 | | 0.222 | |
| *33- 45 months* | | 0.92 (0.08) | | 9.77,1.09 | | 0.340 | |
| *45+ months* | | 0.95 (0.07) | | 9.82,1.10 | | 0.496 | |
|  | |  | |  | |  | |
| IgG CSP (n=52) | |  | |  | |  | |
| *≤ 3-months* | | 3.87 (0.21) | | 3.48,4.30 | | <0.001 | |
| *3-33 months* | | 0.96 (0.004) | | 0.95,0.97 | | <0.001 | |
| *33+ months* | | 0.99 (0.003) | | 0.98,0.99 | | 0.001 | |
|  | |  | |  | |  | |
| IgG NANP (n=52) | |  | |  | |  | |
| *≤ 3-months* | | 3.52 (0.21) | | 3.14,3.95 | | <0.001 | |
| *3-8.5 months* | | 0.90 (0.03) | | 0.85,0.95 | | <0.001 | |
| *8.5-21 months* | | 0.95 (0.01) | | 0.93,0.97 | | <0.001 | |
| *21-33 months* | | 1.00 (0.01) | | 0.98,1.02 | | 0.770 | |
| *33- 45 months* | | 0.95 (0.01) | | 0.92,0.98 | | <0.001 | |
| *45+ months* | | 0.99 (0.01) | | 0.98,1.01 | | 0.477 | |
|  | |  | |  | |  | |
| IgG CT (n=52) | |  | |  | |  | |
| *≤ 3-months* | | 3.48 (0.16) | | 3.17,3.81 | | <0.001 | |
| *3-8.5 months* | | 0.88 (0.02) | | 0.84,0.92 | | <0.001 | |
| *8.5-21 months* | | 0.95 (0.01) | | 0.94,0.97 | | <0.001 | |
| *21+ months* | | 0.98 (0.001) | | 0.987,0.98 | | <0.001 | |
|  | |  | |  | |  | |
| Neutrophil ADRB (n=30) | |  | |  | |  | |
| *≤ 3-months* | | 5.78 (1.24) | | 3.79,8.81 | | <0.001 | |
| *3-8.5 months* | | 0.72 (0.07) | | 0.60,0.88 | | 0.001 | |
| *8.5-21 months* | | 0.96 (0.02) | | 0.93,1.00 | | 0.056 | |
| *21-33 months* | | 0.91 (0.02) | | 0.86,0.96 | | <0.001 | |
| *33+ months* | | 1.02 (0.01) | | 1.00,1.04 | | 0.112 | |
|  | |  | |  | |  | |
| Neutrophil Phagocytosis (n=33) | |  | |  | |  | |
| *≤ 3-months* | | 1.38 (0.09) | | 1.22,1.55 | | <0.001 | |
| *3-21 months* | | 0.96 (0.01) | | 0.95,0.98 | | <0.001 | |
| *21-33 months* | | 0.97 (0.01) | | 0.95,0.98 | | <0.001 | |
| *33- 45 months* | | 0.98 (0.01) | | 0.96,0.99 | | 0.008 | |
| *45+ months* | | 1.00 (0.01) | | 0.98,1.02 | | 0.906 | |
|  | |  | |  | |  | |
| Neutrophil THP1 (n=30) | |  | |  | |  | |
| *≤ 3-months* | | 1.84 (0.07) | | 1.71,1.97 | | <0.001 | |
| *3-33 months* | | 0.96 (0.004) | | 0.95,0.97 | | <0.001 | |
| *33+ months* | | 1.00 (0.002) | | 1.00,1.01 | | 0.330 | |
|  | |  | |  | |  | |
| FcγRIII (n=52) | |  | |  | |  | |
| *≤ 3-months* | | 8.21 (1.02) | | 6.44,10.5 | | <0.001 | |
| *3-8.5 months* | | 0.73 (0.04) | | 0.66,0.80 | | <0.001 | |
| *8.5-21 months* | | 0.91 (0.01) | | 0.89,0.94 | | <0.001 | |
| *21-33 months* | | 0.94 (0.01) | | 0.93,0.96 | | <0.001 | |
| *33+ months* | | 0.95 (0.01) | | 0.94,0.97 | | <0.001 | |
|  | |  | |  | |  | |
| FcγRIIa(n=50) | |  | |  | |  | |
| *≤ 3-months* | | 7.49 (0.83) | | 0.03,9.31 | | <0.001 | |
| *3-8.5 months* | | 0.78 (0.03) | | 0.72,0.85 | | <0.001 | |
| *8.5-21 months* | | 0.90 (0.02) | | 0.87,0.94 | | <0.001 | |
| *21-33 months* | | 0.92 (0.02) | | 0.89,0.96 | | <0.001 | |
| *33+ months* | | 0.95 (0.01) | | 0.94,0.97 | | <0.001 | |
|  | |  | |  | |  | |

^a^ Effects for each factor represent separate generalised linear mixed modelling (GLMM) analyses where each IgG/M subclass and functional factor is regressed on time (per month). Linear splines were used to model the functional form of time. These GLMM’s applied a random intercept for study participant, random slope for time (0-3 months) with an independent covariance structure for the random effects. Coefficients represent the per cent change in IgG/M subclass or functional outcome for a month increase in time. Coefficients less than 1 indicate a (1 – RR * 100) % decrease and above 1 a (RR – 1 * 100) % increase.

^b^ Probability values based on Wald statistics. Statistical significance determined at p < 0.05.

**Table S4: Associations between FcγRIIa and IgG subclass: Rate ratio (RR), Adjusted rate ratio (ARR), associated standard error (s.e.), 95% confidence interval (95% CI) and probability value (p-value) from generalised linear mixed modelling (n=50).**

|  | | *Model A* | | | | | |  | | *Model B* | | | | | |  |  |
| --- | --- | --- | --- | --- | --- | --- | --- | --- | --- | --- | --- | --- | --- | --- | --- | --- | --- |
| *Factor* | | RR^a^ (s.e.) | | 95% CI | | p-value^b^ | |  | | ARR *b* ^a^ (s.e.) | | 95% CI | | p-value^b^ | |  |  |
|  | |  | |  | |  | |  | |  | |  | |  | |  |  |
| IgG1 | | 1.89 (0.19) | | 1.56,2.30 | | <0.001 | |  | | 1.69 (0.16) | | 1.40,2.03 | | <0.001 | |  | |
|  | |  | |  | |  | |  | |  | |  | |  | |  | |
| IgG2 | | 1.46 (0.13) | | 1.22,1.73 | | <0.001 | |  | | 1.05 (0.09) | | 0.89,1.24 | | 0.586 | |  | |
|  | |  | |  | |  | |  | |  | |  | |  | |  | |
| IgG3 | | 1.72 (0.18) | | 1.40,2.12 | | <0.001 | |  | | 1.31 (0.15) | | 1.04,1.64 | | 0.020 | |  | |
|  | |  | |  | |  | |  | |  | |  | |  | |  | |
| IgG4 | | 0.77 (1.87) | | 0.003,90.3 | | 0.915 | |  | | 0.03 (0.04) | | 0.003,0.36 | | 0.005 | |  | |
|  | |  | |  | |  | |  | |  | |  | |  | |  |  |

^a^ Coefficients represent the cent change in participant FcγRIIa for a unit increase in IgG.

^b^ Probability values based on Wald statistics. Statistical significance determined at p < 0.05

Model A = generalised linear mixed modelling (GLMM) analyses where FcγRIIa was regressed on IgG subclass. This GLMM model also included linear splines to model the functional form of time. The GLMM’s applied a random intercept for study participant.

Model B = generalised linear mixed modelling (GLMM) analyses where FcγRIIa was regressed on IgG subclass conditioning on each of the other IgG subclasses to provide an independent association. This GLMM model also included linear splines to model the functional form of time. The GLMM’s applied a random intercept for study participant.

**Table S5: Associations between FcγRIII and IgG subclass: Rate ratio (RR), Adjusted rate ratio (ARR), associated standard error (s.e.), 95% confidence interval (95% CI) and probability value (p-value) from generalised linear mixed modelling (n=52).**

|  | | *Model A* | | | | | |  | | *Model B* | | | | | |  |  |
| --- | --- | --- | --- | --- | --- | --- | --- | --- | --- | --- | --- | --- | --- | --- | --- | --- | --- |
| *Factor* | | RR ^a^ (s.e.) | | 95% CI | | p-value^b^ | |  | | ARR ^a^ (s.e.) | | 95% CI | | p-value^b^ | |  |  |
|  | |  | |  | |  | |  | |  | |  | |  | |  |  |
| IgG1 | | 2.30 (0.39) | | 1.65,3.22 | | <0.001 | |  | | 2.11 (0.42) | | 1.42,3.12 | | <0.001 | |  | |
|  | |  | |  | |  | |  | |  | |  | |  | |  | |
| IgG2 | | 1.37 (0.18) | | 1.06,1.78 | | 0.018 | |  | | 0.86 (0.08) | | 0.73,1.02 | | 0.093 | |  | |
|  | |  | |  | |  | |  | |  | |  | |  | |  | |
| IgG3 | | 1.94 (0.24) | | 1.52, 2.46 | | <0.001 | |  | | 1.45 (0.26) | | 1.03,2.06 | | 0.036 | |  | |
|  | |  | |  | |  | |  | |  | |  | |  | |  | |
| IgG4 | | 0.54 (1.69) | | 0.001,245 | | 0.845 | |  | | 0.02 (0.001) | | 0.002,0.26 | | 0.002 | |  | |
|  | |  | |  | |  | |  | |  | |  | |  | |  |  |

^a^ Coefficients represent the per cent change in participant FcγRIII for a unit increase in IgG.

^b^ Probability values based on Wald statistics. Statistical significance determined at p < 0.05

Model A = generalised linear mixed modelling (GLMM) analyses where FcγRIII was regressed on IgG subclass. This GLMM model also included linear splines to model the functional form of time. The GLMM’s applied a random intercept for study participant.

Model B = generalised linear mixed modelling (GLMM) analyses where FcγRIII was regressed on IgG subclass conditioning on each of the other IgG subclasses to provide an independent association. This GLMM model also included linear splines to model the functional form of time. The GLMM’s applied a random intercept for study participant.

**Table S6: Associations between FcγRIIa, IgG to NANP and IgG to CT: Rate ratio (RR), Adjusted rate ratio (ARR), associated standard error (s.e.), 95% confidence interval (95% CI) and probability value (p-value) from generalised linear mixed modelling (n=52).**

| *Factor* | *RR* ^a^ (s.e.) | 95% CI | p-value^b^ |
| --- | --- | --- | --- |
|  |  |  |  |
| IgG to NANP | 2.88 (0.52) | 2.01,4.11 | <0.001 |
|  |  |  |  |
| IgG to CT | 2.07 (0.32) | 1.52,2.81 | <0.001 |

^a^ Coefficients represent the per cent change in participant FcγRIIa for a unit increase in IgG.

^b^ Probability values based on Wald statistics. Statistical significance determined at *p* < 0.05

Generalised linear mixed modelling (GLMM) was used for analysis and FcγRIIa was regressed on IgG factors. This GLMM model also included linear splines to model the functional form of time. The GLMM’s applied a random intercept for study participant.

**Table S7: Associations between FcγRIII, IgG to NANP and IgG to CT: Rate ratio (RR), Adjusted rate ratio (ARR), associated standard error (s.e.), 95% confidence interval (95% CI) and probability value (p-value) from generalised linear mixed modelling (n=52).**

| *Factor* | RR ^a^ (s.e.) | 95% CI | p-value^b^ |
| --- | --- | --- | --- |
|  |  |  |  |
| IgG to NANP | 3.54 (0.71) | 2.39,5.24 | <0.001 |
|  |  |  |  |
| IgG to CT | 2.96 (0.61) | 1.98,4.43 | <0.001 |

^a^ Coefficients represent the per cent change in participant FcγRIII for a unit increase in IgG.

^b^ Probability values based on Wald statistics. Statistical significance determined at *p* < 0.05

where FcγRIII was regressed on IgG factor. This GLMM model also included linear splines to model the functional form of time. The GLMM’s applied a random intercept for study participant.

**Table S8: Associations between opsonic phagocytosis by neutrophils and IgG subclass: Rate ratio (RR), Adjusted rate ratio (ARR), associated standard error (s.e.), 95% confidence interval (95% CI) and probability value (p-value) from generalised linear mixed modelling (n=33).**

|  | | *Model A* | | | | | |  | | *Model B* | | | | | |  |  |
| --- | --- | --- | --- | --- | --- | --- | --- | --- | --- | --- | --- | --- | --- | --- | --- | --- | --- |
| *Factor* | | RR^a^ (s.e.) | | 95% CI | | p-value^b^ | |  | | ARR^a^ (s.e.) | | 95% CI | | p-value^b^ | |  |  |
|  | |  | |  | |  | |  | |  | |  | |  | |  |  |
| IgG1 | | 1.32 (0.08) | | 1.18,1.48 | | <0.001 | |  | | 1.26 (0.07) | | 1.12,1.41 | | <.001 | |  | |
|  | |  | |  | |  | |  | |  | |  | |  | |  | |
| IgG2 | | 1.16 (0.13) | | 0.93,1.45 | | 0.186 | |  | | 0.88 (0.10) | | 0.71,1.09 | | 0.256 | |  | |
|  | |  | |  | |  | |  | |  | |  | |  | |  | |
| IgG3 | | 1.26 (0.13) | | 1.03,1.54 | | 0.022 | |  | | 1.19 (0.12) | | 0.97,1.46 | | 0.104 | |  | |
|  | |  | |  | |  | |  | |  | |  | |  | |  | |
| IgG4 | | 0.62 (0.63) | | 0.09,4.52 | | 0.639 | |  | | 0.29 (0.25) | | 0.05,1.59 | | 0.152 | |  | |
|  | |  | |  | |  | |  | |  | |  | |  | |  |  |

^a^ Coefficients represent the per cent change in participant Neutrophil Phagocytosis for a unit increase in IgG.

^b^ Probability values based on Wald statistics. Statistical significance determined at *p* < 0.05

Model A = generalised linear mixed modelling (GLMM) analyses where opsonic phagocytosis by neutrophils was regressed on IgG subclass. This GLMM model also included linear splines to model the functional form of time. The GLMM’s applied a random intercept for study participant.

Model B = generalised linear mixed modelling (GLMM) analyses where opsonic phagocytosis by neutrophils was regressed on IgG subclass conditioning on each of the other IgG subclasses to provide an independent association. This GLMM model also included linear splines to model the functional form of time. The GLMM’s applied a random intercept for study participant.

**Table S9: Associations between opsonic phagocytosis by neutrophils, IgG to NANP and IgG to CT: Rate ratio (RR), Adjusted rate ratio (ARR), associated standard error (s.e.), 95% confidence interval (95% CI) and probability value (p-value) from generalised linear mixed modelling (n=33).**

| *Factor* | RR^a^ (s.e.) | 95% CI | p-value^b^ |
| --- | --- | --- | --- |
|  |  |  |  |
| IgG to NANP | 1.49 (0.29) | 1.02,2.17 | 0.039 |
|  |  |  |  |
| IgG to CT | 1.68 (0.25) | 1.25,2.26 | 0.001 |

^a^ Coefficients represent the per cent change in participant Neutrophil Phagocytosis for a unit increase in IgG.

^b^ Probability values based on Wald statistics. Statistical significance determined at *p* < 0.05

Generalised linear mixed modelling (GLMM) was used for analysis and Neutrophil Phagocytosis was regressed on the IgG factors. This GLMM model also included linear splines to model the functional form of time. The GLMM’s applied a random intercept for study participant.

**Table S10: Associations between Neutrophil Phagocytosis, FcγRIII and FcγRIIa binding: Rate ratio (RR), Adjusted rate ratio (ARR), associated standard error (s.e.), 95% confidence interval (95% CI) and probability value (p-value) from generalised linear mixed modelling (n=31).**

| *Factor* | *RR* ^a^ (s.e.) | 95% CI | p-value^b^ |  |
| --- | --- | --- | --- | --- |
|  |  |  |  |  |
| FcγRIII | 1.28 (0.07) | 1.15,1.42 | <0.001 |  |
|  |  |  |  |  |
| FcγRIIa | 1.44 (0.11) | 1.24,1.67 | <0.001 |  |

^a^ Coefficients represent the per cent change in participant Neutrophil Phagocytosis for a unit increase in FcγRIII/FcγRIIa.

^b^ Probability values based on Wald statistics. Statistical significance determined at *p* < 0.05

Generalised linear mixed modelling (GLMM) was used for analysis and Neutrophil Phagocytosis was regressed on FcγRIII/FcγRIIa. This GLMM model also included linear splines to model the functional form of time. The GLMM’s applied a random intercept for study participant.

**Table S11: Associations between opsonic phagocytosis by THP-1 cells and IgG subclass: Rate ratio (RR), Adjusted rate ratio (ARR), associated standard error (s.e.), 95% confidence interval (95% CI) and probability value (p-value) from generalised linear mixed modelling (n=30).**

|  | *Model A* | | |  | *Model B* | | |
| --- | --- | --- | --- | --- | --- | --- | --- |
| *Factor* | RR ^a^ (s.e.) | 95% CI | p-value^b^ |  | ARR^a^ (s.e.) | 95% CI | p-value^b^ |
|  |  |  |  |  |  |  |  |
| IgG1 | 1.29 (0.09) | 1.13,1.47 | <0.001 |  | 1.19 (0.08) | 1.04,1.36 | 0.011 |
|  |  |  |  |  |  |  |  |
| IgG2 | 1.23 (0.12) | 1.02,1.49 | 0.029 |  | 0.93 (0.09) | 0.77,1.12 | 0.417 |
|  |  |  |  |  |  |  |  |
| IgG3 | 1.29 (0.09) | 1.13,1.48 | <0.001 |  | 1.22 (0.07) | 1.09,1.37 | 0.001 |
|  |  |  |  |  |  |  |  |
| IgG4 | 0.24 (0.17) | 0.06,0.95 | 0.042 |  | 0.18 (0.14) | 0.04,0.85 | 0.031 |

^a^ Coefficients represent the percent change in participant Phagocytosis by THP-1 cells for a unit increase in IgG.

^b^ Probability values based on Wald statistics. Statistical significance determined at *p* < 0.05

Model A = generalised linear mixed modelling (GLMM) analyses where opsonic phagocytosis by THP-1 cells was regressed on IgG subclass. This GLMM model also included linear splines to model the functional form of time. The GLMM’s applied a random intercept for study participant.

Model B = generalised linear mixed modelling (GLMM) analyses where opsonic phagocytosis by THP-1 cells was regressed on IgG subclass conditioning on each of the other IgG subclasses to provide an independent association. This GLMM model also included linear splines to model the functional form of time. The GLMM’s applied a random intercept for study participant.
